# Supplementary figures and images for: Evolutionary and functional implications of hypervariable loci within the skin virome
Source: PeerJ. 2017 Feb 7;5:e2959. doi: 10.7717/peerj.2959 (PMC5299996; doi:10.7717/peerj.2959)

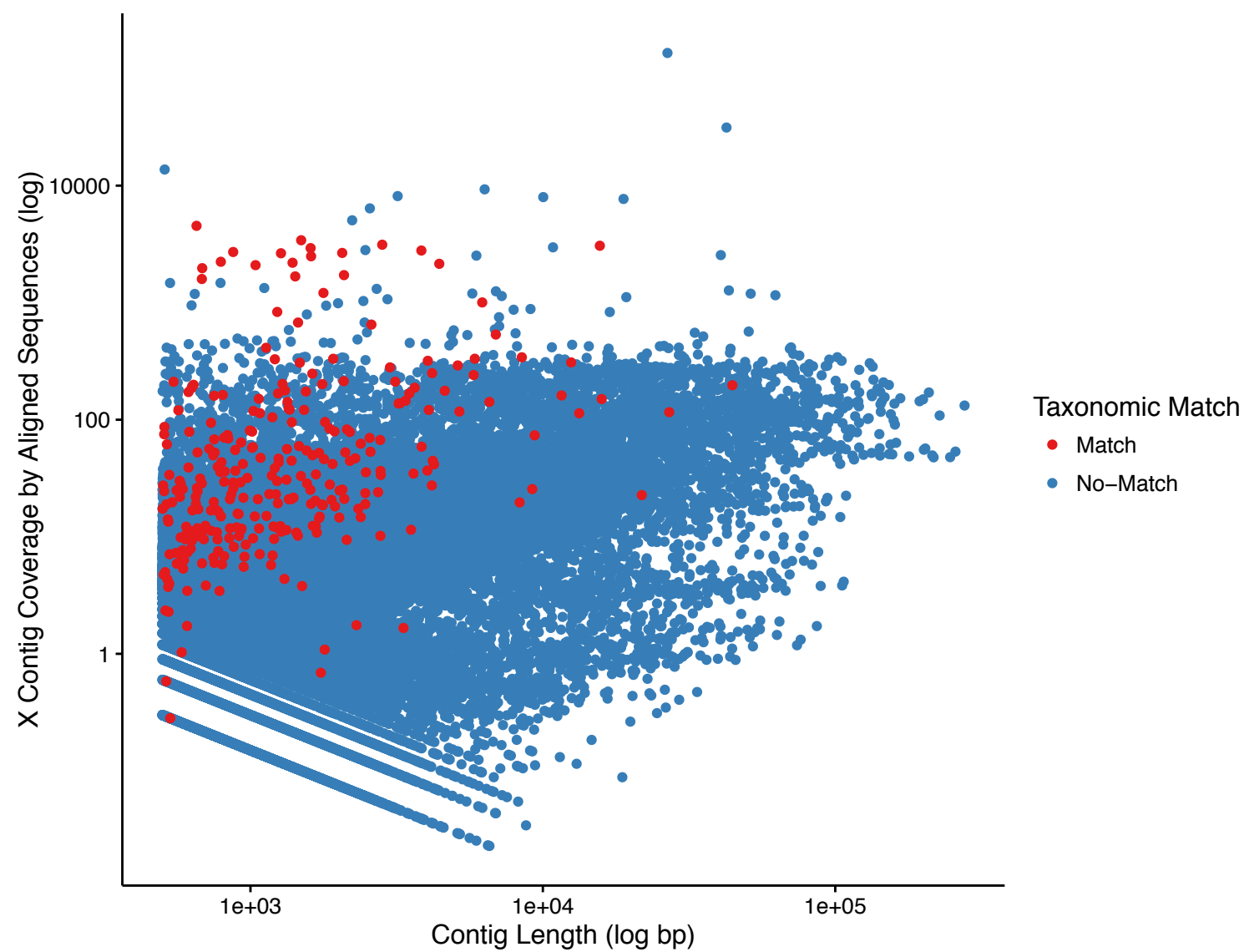

Supplement: Supplemental Information 3 — Total length and sequence coverage of contigs within our study. Those contigs that were included in our targeted, reference-dependent approach (Staphylococcus phage, Propionibacterium phage, Human Papillomavirus) are highlighted in red. [file peerj-05-2959-s003.pdf]

$pN/pS$  Ratio

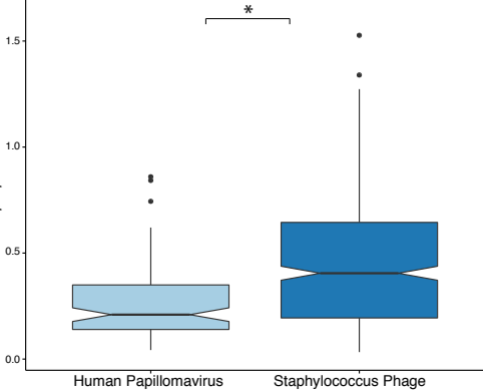

Supplement: Supplemental Information 4 — Notched box plot of the hypervariable loci selective pressures between HPV and Staphylococcus phages. The difference between these populations is significant by Wilcoxon test (p < 0.005; marked by asterisk). [file peerj-05-2959-s004.pdf]

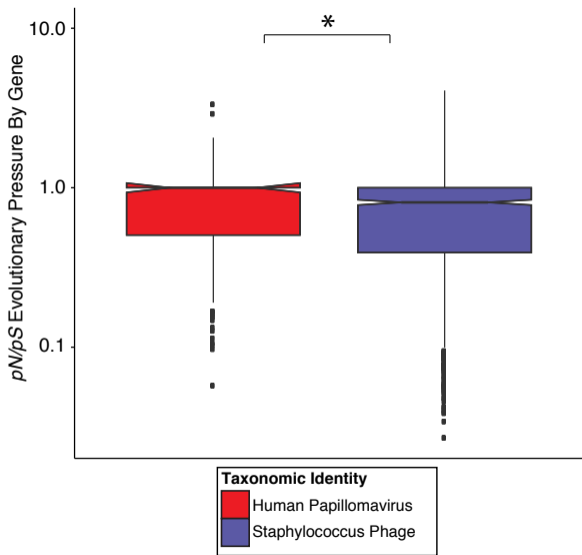

Supplement: Supplemental Information 5 — Box plot of the comprehensive evolutionary pressure on each virus of interest. The y-axis depicts the pN/pS ratio of genes from each virus denoted on the x-axis. Viruses are highlighted in red (HPV) and blue (Staphylococcus phage). The difference between these populations is significant by Wilcoxon test (p < 0.01; marked by asterisk). [file peerj-05-2959-s005.pdf]
